# Changes of the phytoplankton community as symptoms of deterioration of water quality in a shallow lake

**DOI:** 10.1007/s10661-018-6465-1

**Published:** 2018-01-25

**Authors:** Ewa Anna Dembowska, Tomasz Mieszczankin, Paweł Napiórkowski

**Affiliations:** Faculty of Biology and Environmental Protection, Department of Hydrobiology, Nicolaus Copernicus University, Lwowska 1, 87-100 Toruń, Poland

**Keywords:** Shallow lake, Cyanoprokaryota, Phytoplankton community, Clear-water, Turbid state

## Abstract

Covering more than 60% of the lake surface, macrophytes determined the taxonomic composition of phytoplankton. We have found numerous indications of ecological deterioration and an increased trophic level year to year: an increased total number of taxa; a significantly increased number of species of Chlorophyta, Bacillariophyceae and Cyanoprokaryota; a decreased number of Chrysophyceae; increased Nygaard index, and high diversity and variability of phytoplankton functional groups. Within 2 years (2002 and 2003) algal biomass doubled: from 3.616 to 7.968 mg l^−1^. An increased contribution of Chlorococcales and Cyanoprokaryota indicates progressive eutrophication of the lake. The average size of planktonic algae increased, particularly Cyanoprokaryota, where small-celled decreased dramatically and were replaced by large colonies. Cyanoprokaryota remained the dominant group of phytoplankton after 10 years, and the ecosystem of the lake remained in the turbid state. This group of algae had the average biomass 9.734 mg l^−1^, which constituted almost 92% of the total biomass.

## Introduction

Phytoplankton serves as an indicator of trophic state and ecological condition of lakes and rivers (Padisák et al. 2006; Szilágyi et al. 2008; Pasztaleniec and Poniewozik 2010; Phillips et al. 2014; Dembowska and Józefowicz 2015; Dembowska et al. 2015). In shallow lakes, the trophic state (connected with a nutrient level) cannot be considered the only factor determining algal growth.

According to the criterion of water transparency, water bodies are divided into at least two categories. The first includes water bodies with high water transparency, occasionally reaching the bottom, the second—water bodies with low water transparency and regular algal blooms. Scheffer and Jeppesen (1998) supported the hypothesis of “alternative stable states” in shallow eutrophic lakes. A clear-water state is typical of lakes with low concentration of suspended particulate matter (SPM) and low phytoplankton abundance where the bottom is covered with submerged macrophytes. A turbid state is typical of lakes with high concentration of SPM, responsible for low water transparency. Li (1998) distinguished several intermediate stages between the clear-water and turbid states, the stability of which is determined by many factors including nutrient loading and concentration, and the relationships between macrophytes and phytoplankton.

Macrophytes are known to significantly influence the phytoplankton community, decreasing its abundance and biomass, and modifying its species composition (Van Donk and Van de Bund 2002). The following factors limiting growth of algae are the most frequent: competing for nutrients, increasing the pH value, shading the water column and the bottom by macrophytes. Allelopathy can also inhibit the growth of planktonic algae (Hilt and Gross 2008). Organic compounds produced by several macrophyte species cause shifts in the phytoplankton species composition (Scheffer and Jeppesen 1998; Celewicz-Gołdyn 2010; Haroon and Abdel-Aal 2016; Mohamed 2017). In shallow lakes, meteorological factors can also affect phytoplankton biomass and composition (Weithoff et al. 2000), occasionally transforming the entire biological community. Mihaljević et al. (2010) observed that a violent flood may cause shifts from a turbid to a clear-water state.

Zooplankton is yet another limiting factor for phytoplankton population. As a result of intensive filtration, it can significantly reduce the growth of phytoplankton and other elements of the suspended particulate matter, thereby increasing water transparency and improving its quality. It is also an essential element of a trophic chain, especially in shallow lakes (Adamczuk and Kornijów 2011) and can be affected by trophic level fluctuations, anthropogenic influences, and shifts between clear- and turbid-water states.

There is still little information on algal population in shallow clear-water lakes during the transition to the turbid state. The main objective of this study was to prepare a qualitative (i.e., analyzing the species composition) and quantitative (i.e., analyzing the abundance and biomass) assessment of phytoplankton in the lake dominated by macrophytes. The study focuses on plankton community change as an indication of water quality in the lake. Conducted for two periods, the research focused mainly on the lake’s main body. Having observed significant changes in the phytoplankton community within the year to year in the first period, we attempted to determine their nature, paying special attention to the increased trophic level, the deterioration of its ecological status, and a shift to a turbid state.

## Description of the study area

Lake Zielone (53° 33.5′ N; 19° 36.9′ E) is located in the Iława Lake District approximately 5 km southeast of Iława. This typical glacial ribbon lake has an elongated shape and extends longitudinally. It is small (area 20.2 ha) and shallow (maximum depth 2.4 m), it has a maximum length of 1450 m and maximum width of 250 m (Table 1).

**Table 1:** Morphometry of Lake Zielone

Parameter	Value	
Area	20.2	ha
Volume	263	thous. m^3^
Maximum depth	2.4	m
Mean depth	1.3	m
Maximum lenght	1450	m
Maximum width	250	m
Maximum effective length	1010	m
Maximum effective width	250	m
Epilimnion depth^*)^	3.5	m

^*)^According to the Patalas’s formula of epilimnion depth = $4.4\sqrt{D}$

The maximum effective length (1.010 m) determines the wind fetch. The theoretical depth of epilimnion (based on equation $4.4\sqrt{D}$, where *D* stands for the mean value of the maximum effective length and maximum effective width, Patalas 1960) is 3.5 m. Although the lake is surrounded by tall trees reducing the effect of the wind on its surface, the abovementioned parameters indicate increased water dynamics and possible resuspension, especially when the wind blows along the longitudinal axis. Gently sloping area surrounding the lake is covered mostly by mixed forest.

Littoral vegetation was dominated by common reed (*Phragmites australis* (Cav.) Trin. ex Steud) forming an irregular belt. Submerged vegetation consisted primarily of hornwort (*Ceratophyllum demersum* L.), Canadian pond weed (*Elodea canadensis* Rich.), and white and yellow water lilies (*Nymphaea alba* L. and *Nuphar lutea* L.). Macrophytes covered about 60% of the bottom in the year 2002, but in 2012, only 10%.

The species composition of zooplankton with the prevalence of Cladocera *Ceriodaphnia pulchella* (G.O. Sars, 1862), *Diaphanosoma brachyurum* (Liévin, 1848), and Rotifera *Gastropus stylifer* (Imhof, 1891), *Trichocerca cylindrica* (Imhof, 1891), *Trichocerca similis* (Wierzejski 1893), and *Polyarthra dolichoptera* (Idelson, 1925) indicate a pond-like nature of the lake with an abundant community of macrophytes (Cerbin et al. 1999). A majority of dominant species is considered typical of the littoral zone (Rybak and Węgleńska 2003).

## Material and methods

In years 2002 and 2003 (period I), sampling was performed six times a year during the season of active vegetation from April to October, in years 2011 and 2012 (period II)—four times a year.

Physico-chemical parameters including temperature, pH, electrolytic conductivity, and oxygen content were measured using WTW MultiLine P4 multi-parameter probe. Chemical analyzes were performed using standard methods. Total phosphorus (TP) and Kjeldahl nitrogen (KN) concentration were determined after the digestion of the sample with sulfuric acid (Lewandowski et al. 2003; Tecator Digestion Systems®).

Samples for analysis of the phytoplankton species composition were collected with a plankton net No. 25 (by both vertical and horizontal hauls) and preserved with formalin. For quantitative analysis, unconcentrated phytoplankton was collected from beneath the surface at the depth of about 0.5 m and preserved with Lugol’s solution (J in KJ). Algal abundance was determined using Utermöhl method (Utermöhl 1958). Biovolumes of all algal species were calculated using the volumetric method by Hillebrand et al. (1999) and Sun and Liu (2003) and assuming that 1 mm^3^ of algae is equal to 1 mg (Holmes et al. 1969; Elser and Carpenter 1988). A biovolume is presented as biomass (wet weight) per liter (*B*, mg l^−1^).

The evaluation of the aquatic environment based on the species composition of a diatom community was prepared using a list of indicator species by Denys (1991). Diatom species were classified according to their life-forms and trophic requirements. The trophic level of the lake was assessed on the basis of the species composition and biomass using the following coefficients:

- Nygaard Trophic State Index (Hutchinson (1967), after Nygaard (1949)) regarded lakes with ratios of the number of taxa of major algae groups of less than 1.0 as oligotrophic, while those giving the ratios of more than 3.0 as eutrophic.
- Trophic State Index (TSI Carlson 1977; Carlson and Simpson 1996), where a classification is based on calculating a trophic state index (TSI) to dimensionless numbers from 0 to 100 for the following indicators: Secchi disk visibility (TSI_SD_) and total phosphorus (TSI_TP_). The previous indexes allow the comparison of trophic state indicators.
- Functional groups (Reynolds et al. 2002; updated by Padisák et al. (2009)), based on ecological and morphological features of the species which prevail in phytoplankton biomass. Functional groups (Fg) of species illustrate the characteristics of the environment more effectively (e.g., water mixing, trophic level, nutrient deficiency) than single species.

Samples for zooplankton investigation were taken in 2002, during the vegetation season (27 March; 07 May; 21 June; 18 July; 20 September). Zooplankton was caught using a 5-l sampler. Samples were taken at 1 m depth; one sample represented a volume of four samplers (20 l) pooled together and filtered through a plankton net (50 μm). All samples were preserved with Lugol’s solution (Nogrady et al. 1993; Harris et al. 2000). Identification and measurement of zooplankton were performed with a light microscope Nikon Alphaphot YS2, a Panasonic camera, and the MultiScan software for image analysis. Zooplankton was counted under a microscope in a Segdwick-Rafter chamber by the subsample method (McCauley 1984). The abundance of zooplankton was calculated per 1 l^−1^ of water. The rotifers’ and crustaceans’ wet weights were calculated according to the formula of Radwan (2004) and McCauley (1984).

## Results

**Fig. 1:**
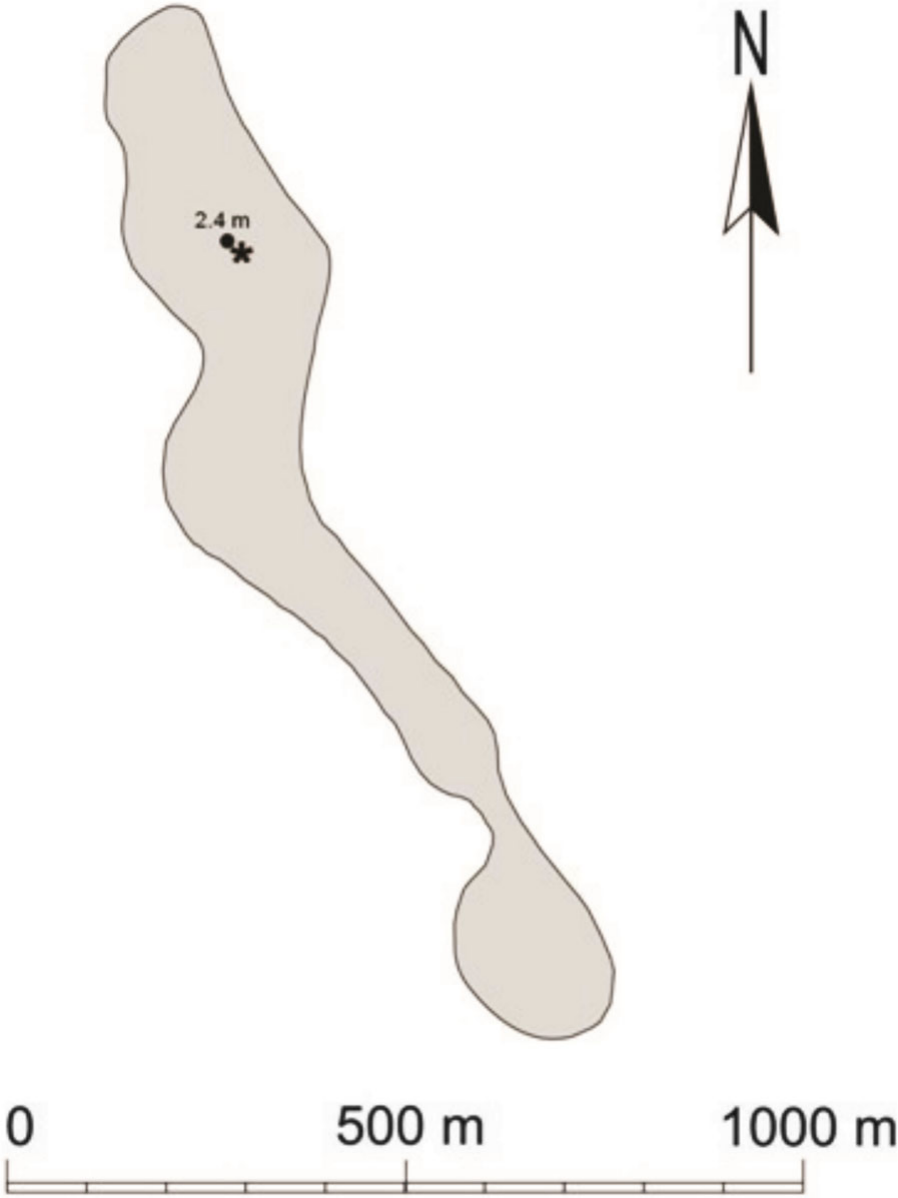
Map of Lake Zielone with the sampling site

**Fig. 2:**
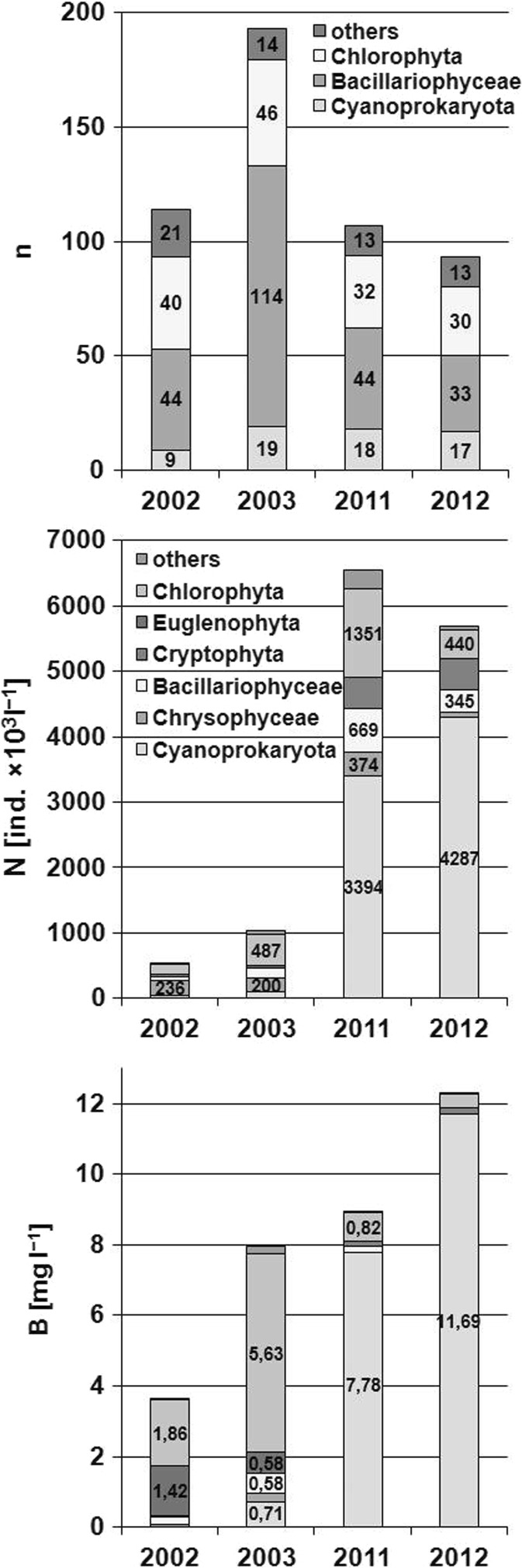
The number of taxa [**n**], mean number of individuals [**N**, 10^3^ind. l^−1^], and mean biomass [**B**, mg l^−1^] of main groups of algae in Lake Zielone

Two hundred eighty-three algal taxa were identified in the phytoplankton of the investigated lake within two periods. The following groups were the most abundant: diatoms (147 taxa), Chlorophytes (79 taxa), and Cyanoprokaryota (28 taxa). Other algal groups were represented by single taxa only (Figs. 1 and 2). One hundred ninety-three taxa were identified during 2003 and only 93 in 2012. The main algal groups had the following percent contribution to the total number of taxa: Bacillariophyceae—39% (2002), 59% (2003), 41% (2011), and 35% (2012); Chlorophyta—35% (2002), 24% (2003), 32% (2011), and 30% (2012); Cyanoprokaryota—8% (2002), 10% (2003), 17% (2011) and 18% (2012).

Nygaard trophic index, based on the algal species composition, was 8.2 (2002) and 9.1 (2003) in the period I and about 10.5—in the period II (2012 and 2013). Some species are typical of eutrophic, some of oligotrophic lakes. The values noted for Lake Zielone were mainly dependent on the number of Cyanoprokaryota (21 taxa), which tolerate high nutrient level.

The average phytoplankton abundance in the first period of investigations was 767 × 10^3^ ind. l^−1^. In 2002, Chrysophyceae were the most abundant (236 × 10^3^ ind. l^−1^), but a year later, Chlorophyta had dominance 487 × 10^3^ ind. l^−1^ (337 × 10^3^ ind. l^−1^ higher than in the previous year, Fig. 2). Bacillariophyceae constituted 9 and 15% of the total algal population in 2002 and 2003, respectively; Cyanoprokaryota constituted 7 and 11% in the subsequent years; and Chrysophyceae—44 and 20%. The average phytoplankton abundance in the second period was 6116 × 10^3^ ind. l^−1^. Cyanoprokaryota were the most abundant, 3840 × 10^3^ ind. l^−1^ (63%). Chlorophyta constituted 15% of the total algal population, Cryptophyta constituted 8%, and diatoms—7%.

**Fig. 3:**
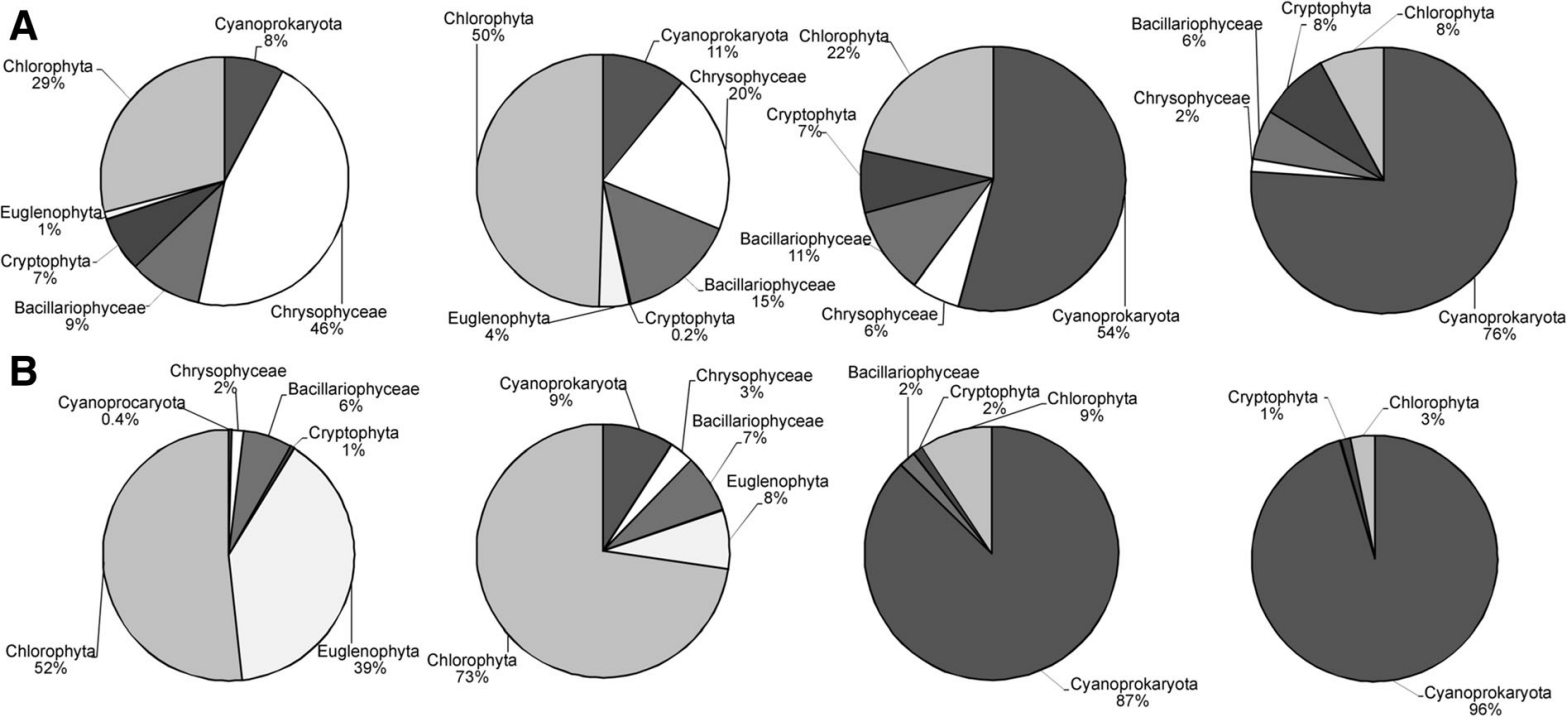
The percentage of main groups of algae (**a** in abundance, **b** in biomass) in 2002 and 2003 years (period I) and 2011 and 2012 (period II) in Lake Zielone

The average phytoplankton biomass in the period I of investigations was 5.792 mg l^−1^; in 2002, it was 3.616 mg l^−1^ and in 2003—7.968 mg l^−1^. Chlorophyta had the highest average biomass in the first year of the research (1.864 mg l^−1^, which constituted 52% of the total biomass) followed by Dinophyta (1.422 mg l^−1^, 39% of the total biomass). The following year, Chlorophyta had the highest contribution (5.633 mg l^−1^, 71%) to the total biomass (Fig. 3). The biomass of Cyanoprokaryota was 0.015 mg l^−1^ (0.4%) and 0.706 mg l^−1^ (9%) in the consecutive years.

Substantial qualitative and quantitative changes in phytoplankton occurred in 2003: a twofold increase in the number of species and the abundance of cyanobacteria was noted in 2003; compared with the previous year, Cyanoprokaryota biomass in 2003 increased nearly 50 times (Fig. 2), making the second highest percent contribution to the total phytoplankton biomass. The following species abounded: *Woronichinia naegeliana* (Unger) Elenkin, *Microcystis aeruginosa* Kutz., *M. wesenbergii* Kom., and *Dolichospermum flos-aquae* (Brébisson ex Bornet & Flahault) Wacklin, Hoffmann et Komárek. The abundance and biomass of Chlorophytes tripled. *Botryococcus braunii*, *Pediastrum boryanum*, *Coelastrum microporum* Nägeli, and *Staurastrum* sp. div developed rapidly. In the phytoplankton biomass almost throughout the entire period I dominated, species belonging to functional groups (Fgs) W2 and F. Chrysophytes (Fg E) were dominant in the spring, in the summer, and the fall—dinoflagellates (Fg Lo) and species belonging to groups J, P, and MP.

The average phytoplankton biomass in the period II of investigations was 10.627 mg l^−1^. Cyanoprokaryota had the highest average biomass 9.734 mg l^−1^, which constituted almost 92% of the total biomass in 2011–2012. During the second period of the study, all the time dominated species from Fgs M (*Microcystis* sp. div.) and K (*Aphanocapsa* sp. div.). In addition, in early spring dominated Fg Y (*Cryptomonas* sp. div.), in the summer, species belonging to Fg H1 (*Dolichospermum flos-aquae*), and F (*Botryococcus braunii*) in late autumn.

**Fig. 4:**
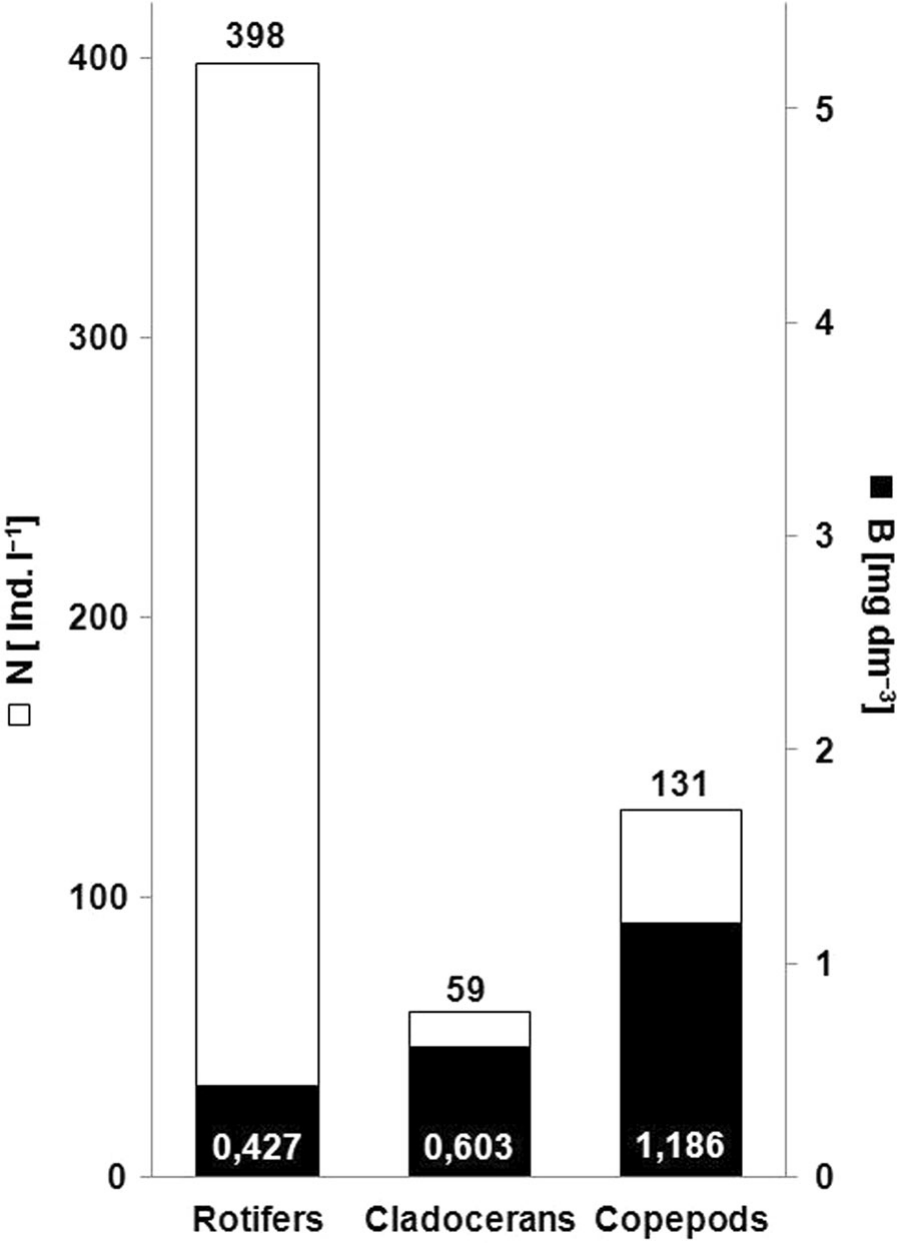
The mean abundance [**N**, ind. l^−1^] and mean biomass [**B**, mg l^−1^] of zooplankton in Lake Zielone in 2002

We identified 52 species in zooplankton. Represented by 42 species, Rotifera constituted 81% of the total zooplankton community. Cladocera, represented by six species, and Copepoda, represented by four species, constituted 12 and 7%, respectively. The average zooplankton abundance was 729 ind. l^−1^, dominated by rotifers (65%), followed by Copepoda (23%) and Cladocera (12%). The dominant species included *Keratella cochlearis* (Rotifera), *Ceriodaphnia pulchella* and *Diaphanosoma brachyurum* (Cladocera), *Gastropus stylifer*, *Trichocerca cylindrica*, *Trichocerca similis*, and *Polyarthra vulgaris* (Rotifera). The zooplankton abundance was on average 588 ind. l^−1^ (Fig. 4). The average zooplankton biomass was 2.730 mg l^−1^. Copepoda had the largest contribution (53%), followed by Cladocera (26%) and Rotifera (21%). The average biomass was 2.216 mg l^−1^.

The Secchi disk (SD) visibility in periods I and II ranged from 0.5 to 2.4 m. The average concentration of SPM was 8.3 and 16.7 mg l^−1^ and total phosphorus—0.088 and 0.113 mg l^−1^, respectively (Table 2). The TSI trophic index based on the SD ranged from 49.4 (2002) and 55.1 (2003) to 66.8 (in 2011) and based on TP—67.5 (2002), 69.8 (2003), and 75.3 (2011).

**Table 2:** Physico-chemical parameters of water in Lake Zielone in two periods: 2002–2003 and 2011–2012

		SD [m]	Temp. [°C]	Oxygen [mg l^−1^]	Oxygen [%]	pH	EC [μS cm^−1^]	SPM [mg l^−1^]	TP [mg l^−1^]	TN^*)^ [mg l^−1^]
2002–2003	Mean	1.5	16.7	11.8	124	8.8	103	8.3	0.088	1.8
	Range	0.9–2.4	5.5–25.6	9.1–15.8	86–140	8.5–9.2	101–104	4.1–14.5	0.06–0.12	1.6–1.9
2011–2012	Mean	0.8	17.6	9.5	113	8.8	81	16.7	0.113	n.d.
	Range	0.5–1.1	5.1–24.9	9.2–12.9	73–159	7.8–10.5	72–109	7.3–32.9	0.06–0.24	

*n.d.* not determined

^*)^TN in the summer

## Discussion

Physico-chemical parameters of water in Lake Zielone indicate relatively low eutrophic level and low electrolytic conductivity. In similar lakes in the Iława Lake District, the average conductivity was about 300 μS cm^−1^. On many occasions, water transparency was high enough for the sunlight to reach the bottom, which is typical of shallow lakes overgrown by macrophytes (Scheffer and Jeppesen 1998). However, in the summer, transparency decreased significantly as a result of intensive phytoplankton growth (Table 2). At the time of sampling, water was generally well oxygenated, and occasionally hypersaturated, which might indicate a high rate of photosynthesis.

Determining the community of organisms inhabiting a water body seems a more accurate method for the assessment of its ecological condition than chemical analysis (Dasí et al. 1998). The dominance of Bacillariophyceae is typical of many Polish eutrophic lakes (e.g., Chudyba and Czaplicka 1993; Luścińska and Rutkowska 1995; Pełechata and Wieścicka 2002). Although the number of Cyanoprokaryota species increased in 2003 (period I of the research), their share in the total number of species did not show significant growth. Ten years later, the share of Cyanoprokaryota in the total number of phytoplankton species doubled. Based on the species composition, the Nygaard trophic state index increased from 8.2 in 2002 to 10.6 in 2012, indicating an increase in the trophy of the lake. A natural consequence of an increased number of cyanobacteria and coccoid Chlorophyta, increased Nygaard index, indicates a slightly elevated trophic level in the period II of the research.

In terms of life-forms, benthic and periphytic diatoms were the most abundant (72% were sedentary). The presence of benthic diatoms of the genera *Navicula*, *Neidium*, *Nitzschia*, *Pinnularia*, and *Stauroneis* in the water column may indicate that higher water dynamics was responsible for their detachment from the surface of macrophytes and from the surface layer of bottom sediments.

Since diatoms are excellent indicators of water quality, they were used to assess the ecological status of Lake Zielone. The species composition indicated a wide adaptability of the identified taxa. In terms of trophic preferences, the most abundant were diatoms tolerating a wide range of trophic levels, from mesotrophic to eutrophic. Next in abundance were species which can live only in eutrophic waters.

Compared to other types of trophic waters, phytoplankton diversity in eutrophic waters is the highest (Lepistö and Rosenström 1998). Year 2003 was marked with the deterioration of the ecological status of the lake (related to its increased trophic level), manifested by an increased total number of taxa; a significantly increased number of species belonging to three major groups of Chlorophyta, Bacillariophycae, and Cyanoprokaryota; a decreased number of taxa belonging to the Chrysophytes; and increased Nygaard index. Moreover, 17 of the identified diatom taxa are characteristic of eutrophic and dystrophic lakes, further indicating an increase in the trophic level. The increase in the number of species according to the theory of intermediate disturbances (Reynolds et al. 1993) was probably due to the slight changes in the ecosystem, for example, transformation of the macrophytes cover or composition.

In the two consecutive years in the period I, algal biomass more than doubled (Fig. 2). In 2002, dominant Chlorophyta and Dinophyta had the highest percent contribution. In 2003, the algal biomass was largely dominated by Chlorophyta (Table 3). The increased contribution of Chlorococcales and Cyanoprokaryota in the biomass is a symptom of progressive eutrophication of the lake (Carlson 1977; Carlson and Simpson 1996; Napiórkowska-Krzebietke and Hutorowicz 2006; Jekatierynczuk-Rudczyk et al. 2014), frequently preceded by the dominance or the bloom of dinoflagellates, which in Lake Zielone occurred in 2002.

**Table 3:** The percentage of the dominant species of algae in total biomass [**B**, mg l^−1^] in the phytoplankton in Lake Zielone in 2002, 2003, 2011, and 2012

	2002			2003			2011			2012		
Month	Taxa	%	**B**	Taxa	%	**B**	Taxa	%	**B**	Taxa	%	**B**
IV	*Peridinium* sp. div	56	1.083	*D. sertularia*	38	2.105				*Cryptomonas erosa*	71	0.667
	*Dinobryon sertularia*	11		*D. cylindricum*	29					*A. holsatica*	18	
	*D. divergens*	10		*Trachelomonas planctonica*	14							
V/VI	*Peridinium* sp. div.	38	0.394	*Staurastrum gracile*	20	8.583				*A. delicatissima*	35	9.500
	*Pediastrum boryanum*	22		*Botryococcus braunii*	18					*Aphanocapsa incerta*	17	
				*P. boryanum*	13					*M. wesenbergii*	12	
				*Staurastrum tetracerum*	12					*Radiocystis geminata*	11	
VII	*Peridinium* sp. div*.*	91	4.668	*Ulnaria ulna*	17	11.283	*Microcystis wesenbergii*	30	13.030	*A. delicatissima*	57	13.132
				*S. tetracerum*	15		*Anabaena flos- aquae*	25		*A. holsatica*	22	
				*Ceratium hirundinella*	12		*Microcystis flos-aqae*	12		*M. wesenbergii*	10	
VIII	*Peridinium* sp. div.	53	5.304	*B. braunii*	14	9.965	*M. wesenbergii*	22	13.935			
	*Botryococcus braunii*	29		*P. boryanum*	10		*Gloeocapsa sanguinea*	22				
				*Coelastrum microporum*	24		*Aphanocapsa holsatica*	15				
				*S. tetracerum*	12							
IX	*Botryococcus braunii*	83	9.547	*B. braunii*	35	9.376	*M. wesenbergii*	44	7.259	*A. holsatica*	46	25.908
	*U. ulna*	13		*P. boryanum*	14		*Microcystis flos-aqae*	15		*M. wesenbergii*	26	
				*Woronichinia naegeliana*	15		*Aphanocapsa delicatissima*	11				
X/XI	*Peridinium* sp. div	54	0.694	*B. braunii*	16	6.491	*A. holsatica*	24	1.576			
	*Kirchneriella* sp.	23		*P. boryanum*	16		*B. braunii*	17				
				*S. gracile*	14		*M. wesenbergii*	13				
				*S. tetracerum*	12							

In the period II (2011–2012), abrupt changes of phytoplankton community were observed. The total biomass of phytoplankton increased to 10.627 mg l^−1^, and Cyanoprokaryota were dominant (92% of the total biomass). The following Cyanoprokaryota species had the highest impact on the total biomass: *Microcystis wesenbergii* (Kom.), *Aphanocapsa holsatica* (Lemm.) Cronb. et Kom., *Aphanocapsa delicatissima* West & G.S. West, and *Microcystis flos-aquae* (Wittr.) Kirchn. The significant contribution of Chroococcales, including potentially toxic species, disturbs ecological stability in this lake.

The remarkable differences in the total number of species, the number of dominant species, the phytoplankton abundance, and the biomass between consecutive years in the period I indicate changes in water quality. In the period II (2011 and 2012), a permanent bloom of water caused by Cyanoprokaryota has been observed. We hypothesize that a dense population of macrophytes may be responsible for restricted algal growth, during the first year of the research. In many eutrophic lakes, macrophytes reduce the phytoplankton biomass thereby increasing water transparency (Lau and Lane 2002). Aquatic vegetation can inhibit phytoplankton biomass through shading, reducing resuspension, limiting nutrients, and allelopathic impact (Scheffer 1999). There can be many reasons that explain the massive development of Cyanobacteria, including changes in the size and the species composition of the macrophyte population. In the second year of the research, *Elodea canadensis* were severely depleted. Cyanobacteria are susceptible to allelopathic influence of submerged plants (Hilt and Gross 2008). According to Kufel et al. (2007), both chlorophytes and cyanobacteria are sensitive to allelochemicals. It has been confirmed in the laboratory that *Stratiotes aloides* inhibits the development of *Scenedesmus obliquus* (Mulderij et al. 2005) and *Chara* inhibits the development of many species of chlorophytes (Mulderij et al. 2003). In 2002 we noted a significantly increased population of dinoflagellates, probably responsible for the restricted growth of cyanobacteria. Experimental studies of Wu et al. (1998), which showed the inhibitory effect of dinoflagellates *Peridinium bipes* Stein on cyanobacteria *M. aeruginosa*, seem to confirm our observation. However, this increased growth of cyanobacteria could have been caused by a higher amount of nutrients, in particular, phosphorus from the bottom sediments and the catchment area.

Compared to other lakes of similar trophic level (Kowalczyk and Radwan 1982; Adamczuk and Kornijów 2011), the zooplankton abundance and biomass in Lake Zielone were relatively low. Zooplankton population depends largely on the density and the species composition of macrophytes (Basu et al. 2000; Kuczyńska-Kippen and Nagengast 2006; Basińska and Kuczyńska-Kippen 2009). The *Elodea* biomass provides food for almost all of macrozoobenthos representatives, including both herbivores and detritivores. By feeding and excreting, these organisms increase the amount of dissolved organic matter and detritus in water, accelerating the development of bacterioplankton and protozoa and the decomposition of organic matter. Abundant algae, bacteria, and small detritus, high quality food for micro- and macrofiltrators (Gliwicz and Rykowska 1992), improved conditions for zooplankton growth. Species prevailing in the phytoplankton biomass in the period I were typical of shallow eutrophic waters, classified into seven functional groups described by Reynolds et al. (2002) and Padisák et al. (2009). Common in small, oligotrophic, base-poor lakes or heterotrophic ponds (Reynolds et al. 2002; Padisák et al. 2009), chrysophytes *Dinobryon* (Fg E) were dominant in April 2002 and 2003. Their low biomass at that time indicates nutrient deficiency (mixotrophy of *Dinobryon*). However, the large biomass of *Trachelomonas planctonica* Swirz. (Fg W2) at that time and *Botryococcus braunii* (Fg F) almost throughout the entire growing season indicates a nutrient level typical of meso- and eutrophic waters. In the summer, we identified dinoflagellates (Fg Lo), typically found in lakes of different sizes, depths, and trophic states. We also recorded species belonging to groups J, P, and MP, found in shallow, strongly mixed, and fertile water bodies. The fall was characterized by the dominance of species typical of shallow and fertile habitats, belonging to the groups listed in the summer.

In the period II, a completely different phytoplankton was found, classified to the group M typical of eutrophic to hypertrophic, small- to medium-sized water bodies and group K recorded in shallow, nutrient-rich water columns. Groups H1 (*Dolichospermum flos-aquae* in the summer), Lo (*Radiocystis geminata* in the spring), and F (*Botryococcus braunii*) i Y (*Cryptomonas erosa*, *C. marssonii*), late autumn and early spring, were dominant periodically.

In natural environment, many factors other than nutrient concentration contribute to the ecological condition of a shallow eutrophic lake. Temporary disturbances and irregularities such as temperature fluctuations and variable mixing intensity (during windy summers) may cause changes in major phytoplankton functional groups and in the dynamics of intermediate states. The results obtained by Asaeda et al. (2002) indicate the possibility of changes leading to increased phytoplankton abundance. Several factors may promote phytoplankton growth, e.g., high temperature contributes to early spring blooms, which shade the lake thus inhibiting macrophyte development. When the amount of available nutrients increases, changes are more distinct and the lake may experience a transition from a clear-water to a turbid state. The relief of these disorders can restore regular biocenosis. The ongoing research focuses not only on phytoplankton community but also on forms and distribution of phosphorus and nitrogen in the water column and the sediments. An assessment of the ecological and trophic status of the lake is to be prepared. More importantly, the future research will be aimed at determining the relationship between the biomass produced by specific algal groups and the quantity and quality of suspended particulate matter.
